# Serum Bicarbonate Deficiency in Dogs with Acute and Chronic Kidney Disease

**DOI:** 10.3390/vetsci10050363

**Published:** 2023-05-19

**Authors:** Ilaria Lippi, Francesca Perondi, Eleonora Gori, Alessio Pierini, Lucrezia Bernicchi, Veronica Marchetti

**Affiliations:** Dipartimento di Scienze Veterinarie, Università di Pisa, 56124 Pisa, Italy

**Keywords:** bicarbonate, dog, AKI, CKD, ACKD, kidney disease, metabolic acidosis

## Abstract

**Simple Summary:**

Serum bicarbonate deficiency is a well-known disorder in humans with chronic kidney disease and can affect the progression and the outcome of the disease. We hypothesized that a similar disorder was present also in dogs affected by both acute and chronic kidney disease. The aim of our retrospective study was to investigate the frequency and severity of bicarbonate deficiency in dogs with AKI, ACKD, or CKD, and its association with the severity of azotaemia and calcium phosphate disorders. Bicarbonate deficiency resulted in a more frequent and severe condition in AKI and ACKD compared to CKD, probably due to a more severe and sudden loss of kidney function. Finally, a significant association was found between the frequency of bicarbonate deficiency and the IRIS grade/stage and calcium phosphate abnormalities.

**Abstract:**

Serum bicarbonate deficiency is a disorder frequently found in human patients with acute (AKI) and chronic (CKD) kidney disease, due to abnormalities in kidney generation and reabsorption of bicarbonate. Although alkali supplementation is usually performed in both humans and veterinary CKD patients, data regarding the frequency of bicarbonate disorders in AKI and CKD dogs are scarce. The aim of the present study is to assess the frequency and the severity of bicarbonate deficiency of dogs affected by AKI, acute chronic kidney disease (ACKD), and CKD, and to investigate its possible association with the IRIS grade/stage as well as with disorders of calcium phosphate metabolism. A retrospective evaluation of the serum biochemical panels of all dogs with diagnoses of AKI, ACKD, and CKD referred to the nephrology and urology service of the Veterinary Teaching Hospital of the University of Pisa, between January 2014 and January 2022, was performed. Bicarbonate deficiency was defined as serum bicarbonate < 22 mmol/L and classified as moderate (between 18 and 22 mmol/L) or severe (<18 mmol/L). Serum bicarbonate deficiency was found in 397/521 dogs (76%), of which 142/397 (36%) showed moderate deficiency, and 255/397 (64%) severe deficiency. Dogs with AKI and ACKD showed a significantly higher frequency of bicarbonate deficiency (*p* = 0.004) and severe forms compared to CKD dogs (*p* = 0.02). In AKI and ACKD dogs, a negative linear correlation was found between serum bicarbonate and serum creatinine, urea, and phosphate. The frequency of bicarbonate deficiency was higher in the later stages of the disease in both AKI (*p* = 0.01), ACKD (*p* = 0.0003), and CKD dogs (*p* = 0.009). Dogs with serum CaxP ≥ 70 mg^2^/dL^2^ showed a higher frequency of bicarbonate deficiency (*p* = 0.01) and showed severe forms (*p* = 0.01) compared to dogs with CaxP < 70 mg^2^/dL^2^. Serum bicarbonate deficiency seems to be a very frequent disorder in both AKI, ACKD, and CKD dogs, with an increasing frequency and severity in more advanced stages of kidney disease. The higher frequency and severity of bicarbonate deficiency in AKI and ACKD may be caused by a more severe and sudden loss of kidney function, or extra-renal factors. Finally, the association between frequency and severity of bicarbonate deficiency and abnormal CaxP may suggest a potential connection between metabolic acidosis and bone mineral disorders.

## 1. Introduction

Under physiological conditions, kidneys play a fundamental role in maintaining the acid–base balance by regulating reabsorption and the production of new bicarbonate in renal tubules. In chronic kidney disease (CKD) patients, the decline in the functional kidney mass is often associated with the development of metabolic acidosis. 

Although the diagnosis of metabolic acidosis should include the assessment of both serum bicarbonate and blood pH, a blood gas analyzer may not be available in clinical practice. Serum bicarbonate is usually included in the biochemistry panel for the evaluation of kidney diseases, and it may be used as a surrogate for the acid–base status. A serum concentration of bicarbonate below 22 mmol/L is commonly considered suggestive of metabolic acidosis. This metabolic disorder is usually considered a frequent finding in both human and veterinary CKD patients, with increasing frequency in more advanced stages of the disease [1,2]. In uremic patients, metabolic acidosis is responsible for a wide spectrum of disorders, including protein malnutrition and bone demineralization [3].

In CKD humans, serum bicarbonate < 22 mmol/L has been associated with higher risk of CKD progression and mortality [4]. Alkali supplementation is usually performed in both veterinary and human CKD patients with bicarbonate deficiency in order to avoid the deleterious effects of metabolic acidosis, especially on muscle mass and bones [1,5]. Data regarding the frequency and degree of bicarbonate deficiency in dogs with acute kidney injury (AKI) and CKD are scarce, especially concerning the earlier stages of the disease.

The aim of the present study is to assess the frequency and the severity of bicarbonate deficiency in dogs affected by AKI, acute on chronic kidney disease (ACKD), and CKD, and to investigate its possible association with the IRIS grade/stage as well as with disorders of calcium phosphate metabolism.

## 2. Materials and Methods

A retrospective database review of all dogs presented to the nephrology and urology services of the Veterinary Teaching Hospital “Mario Modenato” of the University of Pisa with a diagnosis of AKI, ACKD, CKD, between January 2014 and January 2022, was conducted through the electronic database OCIROE. 

The classification of dogs into AKI, ACKD, or CKD was based on historical, clinical, laboratory, and imaging findings. Dogs were classified as AKI if they met the following criteria: (1) acute onset of clinical signs compatible with AKI (vomiting, lethargy, anorexia); (2) absence of abdominal ultrasound findings suggestive of chronic disease (increased renal echogenicity, decreased cortico-medullary differentiation, decreased kidney size or asymmetry, presence of renal cysts); and (3) absence of pre-existing azotemia (serum creatinine < 1.4 mg/dL; or serum SDMA < 18 μg/dL). Dogs with historical, laboratory, and ultrasound findings relating to CKD that had not experienced acute dysfunction of kidney function over the previous 3 months were classified as CKD. Dogs were classified as ACKD if they showed the following criteria: (1) acute onset of clinical signs compatible with AKI, and (2) pre-existing azotemia (serum creatinine > 1.4 mg/dL; or serum SDMA ≥ 18 μg/dL) and/or abdominal ultrasound signs suggestive of chronic disease. 

Serum biochemical panels, including creatinine, urea, SDMA, total calcium, ionized calcium, phosphate, calcium phosphate product (CaxP), and bicarbonate, were included in the study. Blood samples were taken from different venous sites (jugular, cephalic, or saphenous vein) and immediately collected in methacrylate blood tubes (2.5 mL). All the included serum parameters were assessed through the automatic biochemistry analyzer SAT 450 (Assel, Roma, Italia). Within a maximum of 15 min after blood collection, the samples were centrifugated and submitted for analysis.

Serum biochemical panels were excluded from the study in the case of: (1) one or more missing parameters; (2) post-dialysis panels; and (3) dogs already on IV or oral supplementation of sodium bicarbonate. Medical records of the included dogs were reviewed, and data concerning diagnosis (AKI, ACKD, CKD), breed, age, body weight, diet, use of bicarbonate supplementation, and use of a phosphate binder were recorded.

CKD dogs were classified according to serum creatinine and/or serum SDMA in IRIS stage 1, stage 2, stage 3, and stage 4. AKI and ACKD dogs were classified according to serum creatinine in IRIS grade 1, grade 2, grade 3, grade 4, and grade 5 [6]. 

Serum bicarbonate was considered low if <22 mmol/L. The degree of serum bicarbonate deficiency was considered moderate for serum bicarbonate between 18 and 22 mmol/L, and severe for serum bicarbonate <18 mmol/L. Serum CaxP was considered abnormal if ≥70 mg^2^/dL^2^. 

### Statistical Analysis

The distribution pattern of continuous variables was assessed using the Kolmogorov–Smirnov normality test. Since some variables did not show a normal distribution, all continuous variables were presented as median and minimum and maximum values, and the Kruskal–Wallis test was used to compare median values among the study groups (AKI, ACKD, and CKD). Fisher’s test was used to compare the frequency and severity of bicarbonate deficiency among dogs with different diagnoses (AKI, ACKD, CKD), grades, or stages of the disease, and between dogs with normal and abnormal serum CaxP. The Mann–Whitney test was used to compare median serum bicarbonate levels between dogs with normal and abnormal serum CaxP. The Spearman correlation analysis was used to evaluate correlations between serum bicarbonate and serum creatinine, urea, phosphate, total calcium, ionized calcium, and CaxP, in AKI, ACKD, and CKD dogs. Statistical analysis was conducted with GraphPad Prism^®^.

## 3. Results

A total of 610 medical records of dogs affected by AKI (n = 135), ACKD (n = 191), and CKD (n = 284) were retrieved from the electronic database for the considered time period. Eighty-five records were excluded as they referred to post-dialysis blood work, ten records were excluded due to multiple missing data, and nine records were excluded as the dogs were already on IV sodium bicarbonate supplementation. Of the initially found 610 medical records, 521 medical records were included in the study. 

**Figure 1:**
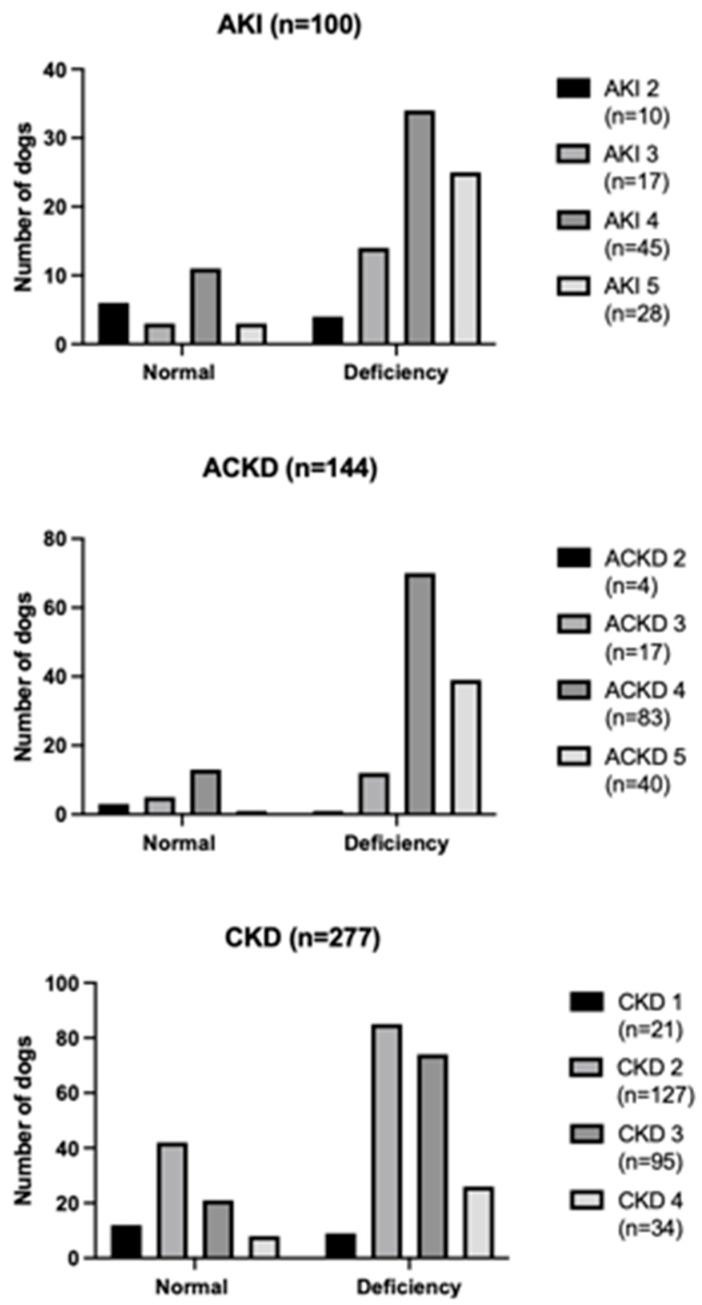
Distribution of bicarbonate status in dogs affected by AKI, ACKD, and CKD at different grades and stages of the disease.

According to diagnosis, medical records were categorized into AKI (n = 100), ACKD (n = 144), and CKD (n = 277). The AKI group was composed of intact females (n = 17), sterilized females (n = 18), intact males (n = 61), neutered males (n = 4), with a median body weight of 15 kg (4.6–40 kg) and a median age of 5 years old (1–17 years old). The ACKD group was composed of intact females (n = 32), sterilized females (n = 55), intact males (n = 50), neutered males (n = 7), with a median body weight of 21 kg (2.9–34 kg) and a median age of 8 years old (1–20 years old). The CKD group was composed of intact females (n = 63), sterilized females (n = 64), intact males (n = 129), neutered males (n = 21), with a median body weight of 16 kg (2.5–30.6 kg) and a median age of 10 years old (1–19 years old). The distribution of bicarbonate status in dogs affected by AKI, ACKD, and CKD at different grades and stages of the disease is reported in Figure 1.

A prescription renal diet was present in 62/100 (62%) of AKI dogs, in 110/144 (76%) of ACKD dogs, and in 260/277 (94%) of CKD dogs. The remaining 38/100 (38%) of AKI dogs, the 34/277 (12%) of ACKD dogs, and the 17/277 (6%) of CKD dogs were on a gastro-intestinal low-fat diet.

Results of the Kruskal–Wallis comparison of the median values of serum creatinine, urea, bicarbonate, total calcium, ionized calcium, phosphate, and CaxP among AKI, ACKD, and CKD dogs, are reported in Table 1.

Serum bicarbonate was available in all the 521 enrolled dogs. Serum bicarbonate deficiency was found in 397/521 dogs (76%). Of the 397 dogs with serum bicarbonate deficiency, 142/397 (36%) showed a moderate deficiency, while 255/397 (64%) showed a severe deficiency. Serum bicarbonate deficiency was found in 77/100 AKI dogs (77%), in 123/144 ACKD dogs (85%), and in 197/277 CKD dogs (71%). AKI dogs with serum bicarbonate deficiency were distributed into grade 2 (n = 4), grade 3 (n = 14), grade 4 (n = 34), and grade 5 (n = 25); ACKD dogs with serum bicarbonate deficiency were distributed into grade 2 (n = 1), grade 3 (n = 13), grade 4 (n = 70), and grade 5 (n = 39); CKD dogs with serum bicarbonate deficiency were distributed into stage 1 (n = 9), stage 2 (n = 85), stage 3 (n = 74), and stage 4 (n = 26). According to the severity of serum bicarbonate deficiency, moderate deficiency was found in 24/77 AKI dogs (31%), 35/123 ACKD dogs (28%), and 83/197 CKD dogs (42%); while severe bicarbonate deficiency was found in 53/77 AKI dogs (69%), 88/123 ACKD dogs (72%), and 114/197 CKD dogs (58%). Results of the comparison in the frequency of serum bicarbonate deficiency and its severity among dogs of the AKI, ACKD, and CKD groups are reported in Table 2.

Serum CaxP was available in 516 dogs. Abnormal serum CaxP was found in 319/516 dogs (62%), and normal serum CaxP was found in 197/516 dogs (38%). Serum bicarbonate deficiency was found in 251/319 dogs with abnormal serum CaxP (79%), and in 136/197 dogs with normal serum CaxP (69%). In dogs with abnormal serum CaxP, moderate deficiency was found in 74/251 (29%) dogs and severe deficiency was found in 177/251 (71%) dogs; while in dogs with normal serum CaxP, moderate deficiency was found in 58/136 (43%) dogs and severe deficiency was found in 78/136 (57%) dogs. Results of the comparison between the frequency of serum bicarbonate deficiency and its severity between dogs with normal and abnormal serum CaxP are reported in Table 2. Dogs with abnormal CaxP showed a significantly lower (*p* < 0.0001) median concentration of serum bicarbonate (16 mmol/L; 3–44 mmol/L) compared to dogs with normal CaxP (19 mmol/L; 3–24 mmol/L).

Results of the Spearman correlation between serum bicarbonate and serum creatinine, urea, phosphate, total calcium, ionized calcium, and CaxP in dogs with AKI, ACKD, or CKD are reported in Table 3.

## 4. Discussion

Serum bicarbonate deficiency was a very common disorder in all three study groups of our population, with an overall frequency of 76%. This was an expected finding, as bicarbonate deficiency and metabolic acidosis have been historically associated with kidney failure in both veterinary and human medicine [1]. In physiologic conditions, kidneys are responsible for the maintenance of acid–base balance by regulating renal generation and supporting the reabsorption of bicarbonate [1,7]. As the majority of urinary bicarbonate (approximately 80%) is reabsorbed at a steady rate in the proximal tubule, an increase in tubular reabsorption of bicarbonate does not commonly represent the most important response to acidosis. The generation of new bicarbonate is mainly achieved in the proximal tubule through ammonia production [2]. Glutamine is the primary substrate for ammoniagenesis in the proximal tubule. At this stage, bicarbonate reabsorption is mediated by a specific transporter (electrogenic sodium bicarbonate cotransporter isoform 1A; NBCe-1 A) [8]. These receptors are present exclusively in the proximal tubule, and they play a key role in bicarbonate reabsorption. Experimental deletion of NBCe-1 A cotransporters in mice has been associated with spontaneous metabolic acidosis, impaired ammonia excretion, and dramatic reduction of serum bicarbonate [9]. Therefore, conditions of proximal tubule injury associated with AKI, ACKD, or CKD may contribute to the development of metabolic acidosis.

In the CKD group, bicarbonate deficiency was found in 71% of dogs, with an increasing frequency in the progression of the IRIS stage. This finding is similar in both human and veterinary medicine, and it may be a consequence of a progressive loss in residual kidney function [1,10]. As kidney function declines, the ability of kidneys to regulate acid–base balance progressively fails. With the progression of CKD, a decrease in the net renal acid excretion occurs, despite an enhancement in the single nephron acid excretion. Although the single nephron activity of acid excretion increases, the remaining nephrons are insufficient to maintain an adequate acid–base balance. The reduction in ammoniagenesis has been directly related to the inability to uptake glutamine at the proximal tubule, and it has been considered responsible for the development of metabolic acidosis in patients with a moderate degree of CKD [1]. In human patients with CKD, as GFR decreases below 15 mL/min/1.73 m^2^, the ability to excrete titratable acids significantly reduces, leading to a worsening of the metabolic acidosis [11]. Therefore, it is not surprising that the majority of dogs at advanced stages of CKD showed bicarbonate deficiency. This trend is in agreement with human medicine, in which the frequency of bicarbonate deficiency significantly increases in CKD stages 4 and 5 [2]. However, the lack of correlation in CKD dogs between serum bicarbonate and creatinine and urea, may suggest mechanisms other than the loss of kidney functions in promoting acidosis. During CKD, serum concentrations of bicarbonate may also be affected by higher or lower consumption of acidic diets and the efficiency of extra-renal mechanisms compensating for metabolic acidosis [12].

Although the majority of dogs at IRIS stage 1 showed normal serum bicarbonate, a relevant percentage of dogs (43%) reported bicarbonate deficiency. This was a very interesting finding, which made us consider bicarbonate deficiency a more frequent disorder than usually thought. A previous study investigating acid–base disorders in cats with CKD showed that metabolic acidosis was a common finding only at later stages of the disease, thus considering metabolic acidosis more as a consequence rather than a cause of progression of CKD [10]. The presence of bicarbonate deficiency at IRIS stage 1 may be a consequence of an enhanced extrarenal generation of acid, rather than the loss of kidney functions. Net acid production contributes to the acid–base balance, and it may be influenced by diet. In human CKD patients, the use of highly acidic diets has been considered as a potential contributor to metabolic acidosis [13]. As all the dogs at IRIS stage 1 were not yet on a prescription renal diet, it is possible that they experienced enhanced net acid production. Finally, we cannot exclude that additional disorders of tubular acid excretion might play a role in reducing serum bicarbonate, as previously documented in CKD humans with obstructive nephropathy, sickle cell nephropathy, and diabetic nephropathy [13]. 

The frequency of dogs with bicarbonate deficiency was significantly higher in dogs with AKI and ACKD compared to CKD. This may be the consequence of a more severe and sudden loss of kidney mass in acute forms. It is possible that the severe and acute reduction in tubular ammoniagenesis, as well as the increase in the filtered acid load, are responsible for a reduction in serum bicarbonate. Besides a severe loss of functional kidney mass, extra-renal factors may contribute to the development of bicarbonate deficiency. Dogs with AKI and ACKD might also show hyperkalemia, primarily as a consequence of inadequate urine production. Hyperkalemia has been proven to cause reversible metabolic acidosis by inhibiting ammonia excretion. Experimental mice models showed that hyperkalemia is responsible for metabolic acidosis in the absence of other known promoting factors, such as reduction of the kidney mass, adrenal insufficiency, or pharmacological inhibition [14]. It should also be noticed that the frequency of dogs on a prescription renal diet was higher in the CKD group compared to the AKI and ACKD groups. In AKI patients, the origin of bicarbonate deficiency is usually considered multifactorial [15]. Among extra-renal causes of metabolic acidosis, gastro-intestinal loss of bicarbonate may play a significant role. Gastro-intestinal signs have been frequently reported in dogs affected by AKI. In particular, the presence of diarrhea has been found in 41% of AKI dogs, with a significantly higher prevalence in non-survivors. During AKI, diarrhea may be a direct consequence of the local effect of uremic toxins, or an indirect consequence of conditions, such as acute pancreatitis or overhydration [16]. Dogs with diarrhea may experience a faster intestinal flow of water and electrolytes, with a lower ability to absorb bicarbonate. Another possible cause of bicarbonate deficiency in our AKI and ACKD dogs may be severe hyperphosphatemia. In our study population, dogs with AKI and ACKD showed significantly higher median values of serum phosphate, compared to dogs with CKD. Moreover, a negative linear correlation was found in both AKI and ACKD dogs between serum bicarbonate and serum phosphate, thus indicating a worsening of serum bicarbonate deficiency with the increase in serum phosphate. This finding may be supported by the promoting role of hyperphosphatemia on metabolic acidosis. In humans, severe hyperphosphatemia has been associated with the generation of acidic compounds and neutralization of bicarbonate [17]. Bicarbonate deficiency seemed also to be associated with the progression of the AKI grade, as a negative linear correlation was found between serum bicarbonate and serum creatinine and urea in both AKI and ACKD dogs. It is plausible that serum bicarbonate deficiency becomes more evident as functioning kidney mass declines. 

Dogs with AKI and ACKD were also characterized by a higher frequency of severe forms of bicarbonate deficiency, compared to dogs with CKD. This difference may have several causes. In veterinary medicine, as well as in human medicine, CKD tends to be a more stable pathological condition. The progression rate of CKD may be highly variable due to the presence/absence of different renal and extra-renal factors [18]. In our cohort, dogs of CKD IRIS stages 1 and 2 were approximately half of the whole CKD population. Dogs within the early stages of CKD are usually characterized by a more stable disease condition, a lower progression rate, and risk of uremic crisis [19]. Therefore, it is possible that they had lower chances to develop severe forms of bicarbonate deficiency. On the contrary, the AKI and ACKD groups were mostly composed of dogs at advanced grades of the disease. The higher frequency of severe forms of bicarbonate deficiency in these two groups of dogs may be due to the abrupt reduction in GFR and loss of functioning kidney mass. Moreover, AKI and ACKD dogs may experience different conditions promoting severe bicarbonate loss, such as severe hyperphosphatemia and gastro-intestinal disorders. Among different causes of bicarbonate deficiency, acute pancreatitis may play a potential role. Acute pancreatitis has been associated with AKI in dogs [20] and with negative outcome in dogs submitted to haemodialysis [21]. In physiological conditions, the pancreatic production of bicarbonate is necessary to prevent the premature activation of pancreatic proteases. Therefore, metabolic acidosis may cause a reduction in the pH and an activation of pancreatic proteases. On the other hand, the presence of acute pancreatitis may promote bicarbonate deficiency by direct mechanisms, such as the loss of pancreatic juice, and by indirect mechanisms, such as the presence of lactic acidosis, due to shock, sepsis, or gastro-intestinal bleeding [22]. 

The presence of metabolic acidosis has been associated with several clinical implications and negative outcomes. During metabolic acidosis, compensatory mechanisms, such as single-nephron ammoniagenesis, may become maladaptive and stimulate kidney fibrosis [2]. Metabolic acidosis has also been identified as an independent risk factor for mortality in CKD patients. CKD people with serum bicarbonate deficiency showed a more rapid decline of kidney function and a higher risk of developing end-stage kidney disease [2]. Among the different clinical implications associated with bicarbonate deficiency, a particular role may be played by calcium phosphate disorders, as bones act as buffers [2]. In our study, dogs with abnormal CaxP showed lower serum values of bicarbonate compared to dogs with normal CaxP. Moreover, the frequency and the severity of bicarbonate deficiency were higher in dogs with disorders of calcium phosphate metabolism. This finding may suggest a connection between calcium phosphate abnormalities and metabolic acidosis. Bicarbonate deficiency may stimulate bone demineralization by different mechanisms. In human CKD patients, reduction in serum bicarbonate has been directly related to the loss of bone density, and metabolic acidosis is supposed to contribute to maintaining calcium phosphate disorders. In AKI and ACKD dogs, a negative linear correlation was found between serum bicarbonate and phosphate, and in ACKD dogs a negative linear correlation was found also between serum bicarbonate and CaxP. These findings may suppose a buffering activity of bones during metabolic acidosis. As serum bicarbonate declines, a release of calcium carbonate and phosphate from bones is stimulated in order to buffer the excess of protons [2]. Besides this mechanism, metabolic acidosis is responsible for an increased activity of osteoclasts, and a reduced activity of osteoblasts, which enhances the loss of bone mineral density [2]. Our findings seemed in line with current evidence in human medicine, wherein AKI is often associated with dysregulation of calcium phosphate metabolism. In these patients, the most frequent abnormalities of mineral metabolism are hypocalcemia, hyperphosphatemia, and hyperparathyroidism [23]. However, in our CKD dogs no statistical correlation was found between serum bicarbonate and different parameters of calcium phosphate metabolism. This was a significant difference compared to human CKD, in which metabolic acidosis and hyperparathyroidism often coexist and contribute to the pathogenesis of bone demineralization. In CKD patients, metabolic acidosis is responsible for an increased response of osteoblast-like cells to PTH, with a stimulation of bone reabsorption [3]. The different behavior of dogs with AKI and ACKD compared to dogs with CKD may be influenced by the lower degree of calcium phosphate dysregulation in the CKD group. Dogs with CKD showed significantly lower values of serum phosphate and CaxP, and significantly higher values of serum bicarbonate and iCa, compared to dogs of the AKI and ACKD groups. This difference may be due to the presence of dogs at very early stages of the disease in the CKD group. Dogs at early stages of CKD have usually milder forms of calcium phosphate disorders and metabolic acidosis. On the other hand, CKD dogs at advanced stages of the disease were likely on a prescription renal diet, which might have beneficial effects on calcium phosphate metabolism. 

The present study has several limitations. Early stages of AKI and ACKD are very difficult to diagnose in a clinical setting, so the frequency of bicarbonate deficiency in these dogs could not be assessed. As venous blood gas analysis was not available for the majority of dogs, blood pH, lactate, and anion gap measurements could not be included in the statistical analysis. These parameters would be of great interest for classifying the kind of metabolic acidosis. Moreover, our study focused on the serum bicarbonate level at the time of presentation to the nephrology service of our hospital. Information regarding the follow-up of serum bicarbonate was present only for a limited number of dogs, as the majority of them had routine follow-ups with the referring veterinarian. The underfilling of vacutainer blood tubes has been proven to cause low bicarbonate readings, regardless of whether samples were analyzed immediately or after 40 min [24]. Although blood tubes were filled with the required volume of blood for all our dogs, the possible interference of the bicarbonate readings cannot be ruled out completely. 

## 5. Conclusions

Serum bicarbonate deficiency was a very frequent disorder in both acute and chronic kidney disease; a frequency which significantly increased with the progression of the IRIS stage/grade. As bicarbonate deficiency was found in a relevant percentage of dogs at IRIS stage 1, it is possible that metabolic acidosis starts very early during CKD. The frequency and severity of bicarbonate deficiency were higher in dogs affected by AKI as well as ACKD, probably due to a more severe and sudden loss of kidney function, or to the presence of extra-renal factors. Finally, dogs with abnormalities of calcium phosphate metabolism showed higher frequency and severity of bicarbonate deficiency, suggesting a potential connection between metabolic acidosis and bone mineral disorders. With the elevated frequency of bicarbonate deficiency, and taking its clinical implications into consideration, serum bicarbonate should be routinely assessed in dogs with acute or chronic kidney disease, regardless of the IRIS grade/stage.

## Figures and Tables

**Table 1:** Kruskal–Wallis comparison of median values of serum creatinine, urea, bicarbonate, total calcium, ionized calcium, phosphate, and CaxP among AKI, ACKD, and CKD dogs.

	Reference Range	AKI (n = 100)	ACKD (n = 144)	CKD (n = 262)	*p*-Value
Cr (mg/dL)	0.6–1.5	7.3 ^a^ (2.0–22.9)	7.6 ^a^ (2.0–23.8)	2.7 ^b^ (1.2–10.2)	<0.0001
Urea (mg/dL)	15–55	263 ^a^ (86–652)	337 ^b^ (88–643)	136 ^c^ (20–658)	<0.0001
Bicarb (mmol/L)	14–28	16.5 ^a^ (3–39)	16 ^a^ (3–44)	19 ^b^ (4–38)	<0.0001
tCa (mg/dL)	8.7–11.8	10.2 ^a^ (6.9–16.6)	10.3 ^a^ (4.2–15)	11 ^b^ (4.5–17.3)	<0.0001
iCa (mmol/L)	1.17–1.48	1.2 ^a^ (0.64–1.91)	1.1 ^a^ (0.21–1.53)	1.32 ^b^ (0.62–2.43)	<0.0001
Phos (mg/dL)	2.5–5.0	11.7 ^a^ (2–22.7)	12.3 ^a^ (3–29.3)	5.4 ^b^ (2.6–23.3)	<0.0001
CaxP (mg^2^/dL^2^)	<70	117.5 ^a^ (17.4–258.5)	126 ^a^ (30.7–237)	59.8 ^b^ (26.3–181.3)	<0.0001

Cr: serum creatinine; Bicarb: serum bicarbonate; tCa: serum total calcium; iCa: serum ionized calcium; Phos: serum phosphate; CaxP: serum calcium phosphate product. Significance during Kruskal–Wallis test is indicated by the *p*-value. Significance during Dunn’s multiple comparison test is indicated by superscript letters. Statistical significance was set for *p* < 0.05.

**Table 2:** (A,B) Fisher’s comparison of frequency and severity of serum bicarbonate deficiency among dogs of the AKI, ACKD, and CKD groups, with particular reference to different grades of AKI and ACKD and different stages of CKD (2A); Fisher’s comparison of frequency and severity of serum bicarbonate deficiency between dogs with serum CaxP < and ≥ 70 mg^2^/dL^2^ (2B).

(A)												
Serum bicarbonate	AKI (n = 100)				ACKD (n = 144)				CKD (n = 277)		*p* value	
Normal	23 (23%)				21 (15%)				80 (29%)		0.004	
Deficiency	77 (77%)				123 (85%)				197 (71%)			
	Moderate		Severe		Moderate		Severe		Moderate	Severe		
	24 (31%)		53 (69%)		35 (28%)		88 (72%)		83 (42%)	114 (58%)	0.02	
	AKI (n = 100)				ACKD (n = 144)				CKD (n = 277)			
IRIS grade/stage	2 (n = 10)	3 (n = 17)	4 (n = 45)	5 (n = 28)	2 (n = 4)	3 (n = 17)	4 (n = 83)	5 (n = 40)	1 (n = 21)	2 (n = 127)	3 (n = 95)	4 (n = 34)
Normal	6 (60%)	3 (18%)	11 (24%)	3 (11%)	3 (75%)	5 (29%)	13 (16%)	1 (2%)	12 (57%)	42 (33%)	21 (22%)	8 (24%)
Deficiency	4 (40%)	14 (82%)	34 (76%)	25 (89%)	1 (25%)	12 (71%)	70 (84%)	39 (98%)	9 (43%)	85 (67%)	74 (78%)	26 (76%)
*p*-value	0.01				0.0003				0.009			
(B)												
Serum bicarbonate	CaxP < 70 mg^2^/dL^2^ (n = 197)						CaxP ≥ 70 mg^2^/dL^2^ (n = 319)					
Normal (n = 129)	61 (47%)						68 (53%)					
Deficiency (n = 387)	136 (69%)						251 (79%)					
*p*-value	0.01											
Degree of deficiency												
Moderate (n = 132)	58 (44%)						74 (56%)					
Severe (n = 255)	78 (30%)						177 (70%)					
*p*-value	0.01											

Serum bicarbonate < 22 mmol/L (bicarbonate deficiency); serum bicarbonate ≥ 22 mmol/L (normal bicarbonate). Degree of serum bicarbonate deficiency was defined as moderate (serum bicarbonate between 18 and 22 mmol/L) or severe (serum bicarbonate <18 mmol/L). Statistical significance was set for *p* < 0.05.

**Table 3:** Spearman correlation analysis of the relationship between serum bicarbonate (mmol/L) and serum creatinine (mg/dL), urea (mg/dL), phosphate (mg/dL), total calcium (mg/dL), ionized calcium (mmol/L), and CaxP (mg^2^/dL^2^) in dogs of the AKI, ACKD, and CKD groups.

Bicarbonate vs.	AKI (n = 100)		ACKD (n = 144)		CKD (n = 277)	
	Spearman r	*p* Value	Spearman r	*p* Value	Spearman r	*p* Value
Creatinine	−0.31	0.001	−0.17	0.03	−0.10	0.07
Urea	−0.29	0.003	0.20	0.01	−0.08	0.14
Phosphate	−0.24	0.01	−0.20	0.01	−0.12	0.05
Total calcium	0.18	0.08	−0.04	0.58	0.01	0.83
Ionized calcium	−0.06	0.50	0.18	0.02	−0.05	0.39
CaxP	−0.16	0.11	−0.19	0.02	−0.10	0.09

Statistical significance was set for *p* < 0.05.

## Data Availability

Data can be retrieved from the electronical database OCIROE of the Veterinary Teaching Hospital “Mario Modenato” of the University of Pisa (Italy).
